# What are end-users’ needs and preferences for a comprehensive e-health program for type 2 diabetes? – A qualitative user preference study

**DOI:** 10.1371/journal.pone.0318876

**Published:** 2025-03-03

**Authors:** Tina Rishaug, Anne-Marie Aas, André Henriksen, Gunnar Hartvigsen, Kåre Inge Birkeland, Eirik Årsand

**Affiliations:** 1 Department of Computer Science, Faculty of Science and Technology, UiT The Arctic University of Norway, Tromsø, Norway; 2 Department of Clinical Service, Faculty of Medicine, University of Oslo, Oslo, Norway; 3 Department of Clinical Service, Division of Medicine, Oslo University Hospital, Oslo, Norway; 4 Department of Transplantation, Faculty of Medicine, University of Oslo, Oslo, Norway; Universiti Putra Malaysia, MALAYSIA

## Abstract

**Introduction:**

Type 2 diabetes (T2D) prevalence is rising, which imposes a significant burden on individuals, healthcare systems, and economies worldwide. Lifestyle factors contribute significantly to the escalating incidence of T2D. Consequently, there is an increasing need for interventions that not only target at-risk populations for prevention but also empower individuals with T2D to achieve better self-management and possibly attain remission through sustained lifestyle modifications. Technological tools may improve health outcomes compared to traditional in-person care, and can include registration of important health parameters, provide follow-up and support, and enhance self-management. The aim of this study was to receive feedback from end-users to inform the development of a comprehensive e-health program focusing on lifestyle modification in pre-diabetes and T2D.

**Methods:**

During eight focus group meetings, sixteen adults with pre-diabetes or T2D from all over Norway informed the study about needs and preferences for an e-health program, including essential functionalities and design choices. A questionnaire and paper prototyping were used to complement the discussions in the focus group meetings.

**Results:**

Lack of necessary diabetes knowledge was common, and education was considered essential for improved self-management. Essential functionalities included registration and overview of several health parameters, long-term follow-up and coaching through communication platforms within the program, automatic data transfer from different devices such as blood glucose monitors and smartwatches, and educational courses. To ensure end-users’ satisfaction with the program and increase motivation for long-term usage, the participants rendered tailoring of desired functionalities and content as crucial.

**Conclusion:**

Based on the findings, a list of recommendations was created, containing the most crucial functionalities and features to include when developing e-health and/or m-health tools for people with pre-diabetes and T2D. Future work should include health care personnel to explore their needs and preferences, and ways such an e-health program may enhance patient interaction without increasing workload and resource use.

## Introduction

The prevalence of diabetes is increasing worldwide, and it is estimated that up to 340 000 have type 2 diabetes (T2D) in Norway [1]. Additionally, many have pre-diabetes, a condition defined as elevated blood glucose levels beyond normal values but not meeting the requirements for a diabetes diagnosis [2]. T2D, in combination with other comorbidities such as obesity, can affect morbidity, mortality, and quality of life. Furthermore, the economic costs associated with T2D in Norway were estimated to more than €4 billion in 2019, equalling more than €12.000 per diagnosed patient per year [3]. Early diagnosis and intervention are crucial for successful lifestyle interventions and improved long-term health outcomes, and individuals diagnosed early have a better chance of achieving this compared to those who have lived with the disease without a diagnosis for years [4].

Both national and international guidelines recommend lifestyle intervention, including a healthy diet and increased physical activity, as the primary treatment for newly diagnosed T2D [5–7]. Several studies have demonstrated the effectiveness of moderate lifestyle intervention on reducing disease incidence and improving metabolic outcomes in T2D [8,9]. More intensive lifestyle intervention may even induce remission of T2D [10–13]. Still, lifestyle guidance and follow-up offered to patients in Norwegian healthcare today are commonly insufficient in reaching treatment goals for blood glucose control and other risk factors associated with T2D. Currently, there are no models for lifestyle intervention that leverage the full potential of such treatment. Furthermore, general practitioners (GPs) do not have the capacity and means to provide the comprehensive follow-up needed to aid patients in diabetes care and self-management [14].

Digital technologies in health, including telemedicine, have become increasingly popular in recent years. Telemedicine use has rapidly increased due to its ability to improve health, reduce health inequalities, provide access to health services, and achieve many other important health-related outcomes [15,16]. Electronic health (e-health) and mobile health (m-health) are other examples of approaches used to achieve such health outcomes using digital technologies. E-health includes the use of technologies such as apps, websites, wearable devices, and telemedicine, and can provide healthcare remotely to patients [17]. M-health, a component of e-health, is defined by the World Health Organization (WHO) as *“medical and public health practices supported by mobile devices, such as mobile phones, patient monitoring devices, personal digital assistants, and other wireless devices*” [18]. This can, according to the WHO, include the use of short messaging service (SMS), third and fourth generations of mobile telecommunications (3G and 4G systems), global positioning system (GPS), and Bluetooth technology [18].

Technological tools such as mobile-based applications can aid in, and potentially increase, self-management and follow-up of people with T2D [19–22]. Such tools have demonstrated comparable or even improved health outcomes compared to traditional in-person care [19–23]. This is partly due to the possibility for remote guidance through video consultations, which may reduce costs and reach out to more people (e.g., rural populations) [24]. The use of mobile technology can also facilitate registration of diet, physical activity, body weight, and blood glucose values. However, there are few m-health tools available today that offer comprehensive lifestyle intervention aiming for long-term prevention, improved self-management, and remission of T2D, integrating tailoring to the individual user.

To achieve the goal of developing effective digital tools for different patient populations and diseases, it is essential to include end-users in the process through co-design. This often involves an iterative process in which users are able to engage in all phases of the design, such as development, testing, and retesting [25,26]. The co-design process aims to ensure that users’ preferences and other important aspects are taken into account to facilitate long-term usage [27].

This user preference study aims to inform the development of a comprehensive e-health program for T2D, developed in collaboration with our industry partner ABEL Technologies AS, by investigating end-user preferences and suggestions for implementing a successful solution that promotes long-term usage. The aim is that the e-health program will contain functionalities for registration and management of important health-related parameters (e.g., diet, physical activity, blood glucose levels, sleep, menstrual cycle), a digital platform for personal coaching and follow-up, and educational courses delivered by trained staff.

## Methods

Eligible participants were Norwegian-speaking adults (≥ 18 years) with either pre-diabetes or T2D, who agreed to attend physical or online meetings with up to seven other participants. Participation required access to a computer or smartphone with Microsoft Teams installed.

To gather information on topics related to pre-diabetes and T2D, we used focus group meetings, where participants were able to meet and discuss in groups. Initially, we aimed to recruit approximately 30 participants (estimating a 20% drop-out rate), to create separate groups for pre-diabetes and T2D. However, since we were mostly able to recruit people with T2D, no separate pre-diabetes groups were created. In qualitative research smaller sample sizes are common, including between five and 20 informants to reach saturation. This also depends on the goal (e.g., aiming for homogeneity or maximum variation in the sample) [28]. Our final sample consisted of 16 participants.

We created four focus groups to investigate and discuss preferences and design ideas for a new, comprehensive e-health program for T2D. We allocated the included participants to suitable groups based on their location, where two groups including people in the Oslo and Tromsø regions were invited to participate in physical meetings at Oslo University Hospital and UiT The Arctic University of Norway, respectively. Participants located elsewhere were invited to attend online via Microsoft Teams, resulting in two separate online groups. All participants were asked to attend two meetings each, resulting in a total of eight focus group meetings.

In the end of the meeting series, we experienced that the discussions and answers provided, yielded no further information on the topics we wanted to explore. Therefore, we concluded that no further meetings were necessary.

### Recruitment

We used snowball sampling to recruit participants. Recruitment took place from 19.09.2022 to 18.01.2023. A flyer (S1 Appendix) with information about the study and inclusion criteria was distributed by The University Hospital of North Norway (UNN), Oslo University Hospital (OUH), the Norwegian Diabetes Association (on their Facebook page, website and to local members in the region via email), a Facebook group for diabetes (‘*For oss med diabetes*’), and by the project colleagues on Facebook and LinkedIn.

Potential participants were invited to read more about the study and register by scanning a quick response (QR) code or following a link forwarding them to a secure survey solution developed and hosted by the University of Oslo: Nettskjema [29]. Alternatively, they could send an email directly to the researcher responsible for recruitment (without including any personal or sensitive information). Informed written consent (S2 Appendix), which the participants had to sign before signing up for the study, was collected in the same online form. If registration was done directly by email, we provided the participant with the online Nettskjema form to provide their consent.

The recruitment form was designed using Nettskjema [29]. Questions included participants’ email address, phone number, age, level of education, diagnosis (pre-diabetes or T2D), and which city they lived in or near (Oslo, Tromsø, or neither). Information about city was used to allocate participants to suitable groups.

We contacted every registered person via telephone to discuss their eligibility and the following issues were considered relevant for participation: 1) whether they had illnesses or diseases preventing them from consuming a normal diet, or being physically active (e.g., severe heart or lung disease), 2) whether they had illnesses or diseases causing problems interacting with smartphones (e.g., reduced vision or motor skills), 3) whether they had reduced hearing, and 4) if they had any other relevant issues potentially affecting their participation.

A total of 27 potential participants registered for the study, where five dropped out before the first meeting; one due to family issues, and four were not reachable after completing the informed consent form and registering for the study. Six participants became ill before the scheduled meetings and did not attend.

Sixteen participants were recruited and divided into the four focus groups, where each group consisted of 3–5 participants.

### Focus group meetings

We conducted a total of eight focus group meetings between November 2022 and March 2023. Since the focus group meetings were scheduled in the evening, we split the meeting into two separate meetings to avoid taking too much of the participant’s time for each meeting. All participants were asked to attend both. Each focus group meeting lasted approximately two hours, with a break after approximately one hour. Each group consisted of 4–5 participants and two or three researchers. In all meetings everyone introduced themselves and were given name tags. Semi-structured interview guides (S3 and S4 Appendix) were used in all meetings.

The first meeting started with a short power point presentation about the project. A subsequent discussion was conducted, with focus on participants’ personal experiences living with pre-diabetes or T2D, and their experience using m-health and/or e-health tools for health management (e.g., apps and activity trackers). At the end of the meeting, a demonstration of a continuous glucose monitoring (CGM) device (FreeStyle Libre 2, Abbott Diabetes Care Inc., California, USA) was performed with the intention of understanding whether such a device was wanted by patients with pre-diabetes or T2D. To improve discussions in the following meeting, participants were asked to use a health app of choice until then, and note what they found helpful, interesting, and motivating about it.

The second, and final, focus group meeting started with discussions about motivation and coping aspects when using e-health tools, what functionalities should be included to achieve this, how users would like to receive follow-up and coaching in the e-health program, and discussions about other functionalities such as reminders. In the latter part of this meeting, we explored ideas and thoughts on an educational component planned as a part of the e-health program, followed by a paper-prototyping session. All participants were also invited to take part in testing the prototype of the e-health program, when it is developed.

All meetings were recorded, except the first part of the first meeting, where participants got a short introduction to the study and its aims and where they could share personal stories, get to know each other and feel more comfortable and safe sharing opinions later in the meetings. We used two digital voice recording devices (Olympus WS-853, Olympus Corporation, Tokyo, Japan) for the recording. Participants could ask for statements to be erased from the recording (i.e., not be included in the transcript), or to pause the recording if sharing personal/sensitive information.

### Questionnaire

Between the two focus group meetings the participants were asked to answer a questionnaire (in paper or digital format using Nettskjema [29]). The project team developed the questionnaire to gather information on each end-user’s demographics, needs, and preferences. It included questions about age, gender, diagnosis, and their use of e-health tools, such as tracking health parameters, sharing information with a coach, and follow-up. The full questionnaire can be found in S5 Appendix.

### Paper-prototyping

In the second (final) meeting, a paper-prototyping session took place aiming to visually explore how users want an e-health program to look like (e.g., colours and icons), including required functionalities. This will subsequently be used in further development of the e-health program. Online participants were provided with the necessary equipment via mail (papers, cut-outs of icons and text boxes, sticky tacks, and an instruction form) before the meeting and were asked to have pencils and scissors available. Participants were instructed to insert icons, write and/or draw in the prototypes to present how they would like an e-health program to look like. The prototypes were collected by the researchers in the physical meetings, whereas the online participants took pictures of them and sent them to the researchers afterwards.

### Storage and use of collected information

All information about participants, including name, email address, age, and diagnosis, was stored together with the audio recordings and paper prototypes in a locked cabinet at UiT The Arctic University of Norway, where only two researchers had access. The key to the cabinet was stored in a separate room.

### Transcription of audio recordings

Audio recordings were transcribed verbatim using Microsoft Word on an offline computer not accessible to others and handled by a single researcher. After transcription, the audio recordings were deleted from the recording devices, and the transcripts were saved on encrypted memory sticks (Flash Padlock 3, Corsair Components Inc., California, USA), stored with the other information described above. To ensure confidentiality, participants were de-identified in the transcripts.

### Thematic analysis

Thematic analysis [30] was used to identify patterns within the transcripts. As the aim of the study was to explore specific needs and preferences from the study sample for use in development of our e-health program, we primarily used a deductive approach. This was then supplemented with an inductive approach as codes and themes emerged from the transcripts during the analysis process. After the initial text analysis and familiarization with the dataset was done, we identified 139 codes within the transcripts, which were grouped into initial topics based on the interview guide. Subsequently, after an iterative approach involving meetings with co-authors discussing how to merge codes, we created the five following themes: 1) *Motivational aspects to facilitate self-management and long-term usage,* 2) *Educational content and learning (with sub-theme Living with prediabetes or type 2 diabetes),* 3) *Communication platforms,* 4) *Functionalities and content of the app as an interactive tool,* and 5) *Tailoring.*

The questionnaire results were not included in the thematic analysis performed in this study, but the most important findings have been included in each theme’s subchapters, described below.

### Ethical considerations

After consulting the data protection officer at UiT The Arctic University of Norway, an application to Sikt (*Norwegian Agency for Shared Services in Education and Research* [Reference number: 963427]) was submitted. Sikt concluded that there was a need to develop an internal data management plan and conduct a risk assessment, both of which were carried out and approved by UiT The Arctic University of Norway. Additionally, an assessment to the Regional Committees for Medical and Healthcare Research Ethics (REK [application ID: 474201]) was made, but the study was found exempt of their approval.

## Results

### Demographics

A total of 16 participants (12 males) attended one or both meetings, with age ranging from 30-79 years. Two participants had pre-diabetes, and 14 had a T2D diagnosis. The study sample represented diverse educational levels, where seven had finished high school or equivalent, four started but did not achieve a degree, one achieved a two-year degree, and five achieved a higher educational degree (bachelor’s or higher).

Thirteen participants completed the questionnaire, including one female who was unable to attend the focus group meetings but submitted the questionnaire online.

From the discussions, it was clear that most participants felt comfortable using technological tools such as health apps or other health monitors, such as smartwatches and blood glucose meters. Some participants also stated that using several apps was a part of their everyday life. However, some participants were not familiar with apps or technological tools beyond owning a smartphone or a pedometer. Despite this, all participants expressed a desire for a tool that could aid them in, and enhance self-management of, living with pre-diabetes or T2D.

### Main themes emerging from the focus group meetings

Through the thematic analysis process, we identified the following five main themes:

1. *Motivational aspects to facilitate self-management and long-term usage,* which refers to motivational aspects of the e-health program that contribute to increased self-management and long-term usage, such as use of historical graphs and rewards. Motivation is the most important aspect to maintain behaviour, or in this context, use of the e-health program in the long-term.
2. *Educational content and learning,* which refers to content that increases the user’s knowledge about diabetes and related topics. Educational content can be presented by professionals within different fields such as diabetes, nutrition, and may be conducted as interactive webinars, lectures, quizzes, and similar. A specifically important sub-theme within this theme, *living with pre-diabetes or type 2 diabetes,* refers to concerns or difficulties living with the disease, or the risk of developing the disease, and in which education and learning may lessen these concerns and increase self-management for the user.
3. *Communication platforms,* which refers to systems and tools where the user can receive follow-up (from assigned coaches within the e-health program) and have contact with peers, family, or friends. In addition, educational courses may be arranged on these platforms.
4. *Functionalities and content of the app as an interactive tool*, which refers to functionalities and content including registration of health parameters or goal setting.
5. *Tailoring*, which refers to the possibility to adjust and personalize the e-health program to the individual user and may include changing the frequency of reminders, types of data to monitor, feedback types, and other functionalities.

#### Theme 1: Motivational aspects to facilitate self-management and long-term usage.

To increase motivation, participants wanted the e-health program to be easy to use, require little to no manual input (see Quote 1 (Q1)), and provide information about their progress via historical graphs for all health parameters (e.g., changes in body weight and physical activity level) (see Q2). This would also allow them to act if experiencing setbacks in achieving specific goals or progress (see Q3 and Q4).

*“It should be simple. It should give me quick feedback. Things that take more than five seconds take too much time.” Q1 (Female 50-59 years, T2D)*

*“Progress. If you follow it [the graph] and see that there is progress. That’s the motivation.” Q2 (Male, 50-59 years, T2D)*

*«Green graph when it [the blood sugar] decreases, and if it increases, then it turns red. Then you’ll see that you need to get it together.» Q3 (Male, 60-69 years, T2D)*

*“You know when you’ve cheated with your exercise and diet. You’ll get it black on white, or red on white in the graph, and you can see that you haven’t achieved your goals. You get the historical progress, and I think that is really interesting … You have to learn from your history, and not make the same mistakes.” Q4 (Male, 70-79 years, T2D)*

Personal coaches were viewed as interesting and important, where many wanted feedback from their coach discussing with them what to do next and getting support while making the necessary changes (see Q5). To avoid overwhelming and demotivating the user, the coach should provide feedback in a gradual manner.

*“And it’s “we”, it’s not “me”. And that “we”-thing when it comes to my disease … then there’s someone to talk to about it.” Q5 (Female, 50-59 years, T2D)*

Ease-of-use also includes automatic data transfer from sensor devices and was pointed out as one of the most important features during several focus group meetings (see Q6 and Q7). Rewards and reinforcement/positive feedback was highlighted as important to maintain motivation over time and increases joy and self-management. Furthermore, the participants wanted relevant education to enhance knowledge about their own disease and thereby motivate and provide a feeling of better self-management. Moreover, goal setting and subsequently achieving those goals can increase motivation and usage in the long-term, as well as motivational videos available in the app.

*“What I view as important, is that it can work together with your smartphone, that the activity the phone registers can be put into the app. That would be important for me.” Q6 (Male, 60-69 years, T2D)*

*“Transfer automatically. So that I don’t have to register it, because then I don’t have to think about anything.” Q7 (Male, 60-69 years, T2D)*

Regarding frequency of use, participants who were already using one or more health apps said that using an e-health program every day was acceptable. Participants not using any apps claimed that frequency was too difficult to maintain over time, but they would use the program several days a week or at least weekly. Especially registration of dietary intake seemed to be too cumbersome and time consuming for some to do daily. Hence, the diet functionalities should be easy to use to ensure that users are satisfied and will use it frequently.

From the questionnaire it was confirmed that the e-health program should be simple, understandable, easy to use, provide integration with other devices in an all-in-one solution (e.g., smartwatches and blood glucose meters), show progress, and give an overview of selected health parameters.

#### Theme 2: Educational content and learning.

Education and learning were stated as particularly important. Participants wished to learn and understand their disease, as many experienced not receiving the necessary information at the time of diagnosis and that they were left mostly to themselves (see Q8). Such learning also raises awareness and may aid them in making better decisions for their health, e.g., eating healthier. Participants expressed a desire to learn about factors associated with T2D, especially blood glucose and how it is affected by, e.g., food, activities, and medications, as well as general health. Many perceived information provided by government/authorities (e.g., dietary recommendations) to be confusing and not specific for T2D (see Q9). As diet is specifically important in T2D, most participants wished to learn more about this, as they were not provided with this information when diagnosed.

*“I’m left to myself. There is no one helping me as much as I was hoping for.” Q8 (Male, 50-59 years, pre-diabetes)*

*“Yes, the recommendations you get are so very general. Like this, live healthy, eat healthy. Oh well.” Q9 (Male, 50-59 years, T2D)*

Participants wanted both digital and physical educational courses, where physical meetings allow them to discuss and share experiences more freely with other participants, whereas digital meetings (e.g., interactive webinars) are more practical and may recruit people who are more shy or not comfortable raising questions in a physical space. During courses, they wanted two-way communication with the possibility to ask the lecturer questions and discuss along the way, and they wanted a limited number of participants for the physical meetings. Furthermore, both online lectures and quizzes were acceptable forms of education for many participants, and both introductory courses and advanced courses were requested.

Many pointed out the importance of including family members in the educational components (see Q10) to increase their understanding of the disease. Furthermore, finding trustworthy information from reliable sources was difficult (see Q11), and they wanted the opportunity to search for, or be provided with, such information in the app. Some also requested a portal for finding suitable food recipes. After learning new things, participants pointed out the importance of receiving practical tips on how to implement the information in their daily lives.

*“That the family had understanding for why we needed to do the big changes we had to do back then. And that no diabetes is the same.” Q10 (Female, 50-59 years, T2D)*

*“One can always use Google, but there’s a lot of strange stuff out there. If there was an app with information one could trust, that is a bit more quality assured. So that you know that professionals are behind it, that it’s trustworthy.” Q11 (Male, 30-39 years, T2D)*

***Sub-theme: Living with pre-diabetes or type 2 diabetes:*** Participants expressed several concerns about living with pre-diabetes and T2D. Stigma exists in society, where many feel blamed for developing the disease due to unhealthy lifestyle choices (see Q12), while other important contributing factors, such as genetic predisposition, are left out. Hence, many feel ashamed and want to hide their disease, or experience loneliness because others do not understand their disease. One participant stressed the importance of addressing such taboos in the e-health program to better handle these negative feelings.

*“They feel taboo, that they have […] lived in a bad way, and that’s not right at all.” Q12 (Male, 60-69 years, T2D)*

Furthermore, many pointed out feeling exhausted and tired from the disease and frustrated over not receiving the necessary care and support. Those defined as “well-regulated” by health care professionals experienced not receiving help despite asking for it. One participant stated that living with T2D equals to living according to a consequence that has not yet occurred (see Q13). In contrast, some claimed to live a mostly normal life, only experiencing smaller complications such as glucose spikes or difficulties identifying suitable food/meals while traveling. Importantly, participants requested increased knowledge about T2D, including what causes elevated or lowered glucose levels, what foods to choose, factors associated with T2D, and general information about health and which factors contribute to the development of the disease. Providing such information could potentially reduce the burden of living with T2D.

*“The biggest challenge is the potential [negative consequences] of diabetes. You must act according to a consequence that has not yet occurred. One can lose one’s vision, lose a leg, right? The potential is huge.” Q13 (Male, 50-59 years, T2D)*

The questionnaire provided information about the most important aspects of the users’ everyday life and disease. Eleven out of 13 participants confirmed that having a more stable blood glucose, or regulating their blood glucose better, was important. Eight participants aimed for weight loss and increasing their physical activity levels. Seven participants wanted to eat healthier and more varied. And finally, two wanted to enhance their sleep and feel better (enhanced mood, joy, energy).

#### Theme 3: Communication platforms.

Regarding the use of coaches for follow-up, many pointed out the importance of receiving support from a diabetes-trained coach, as this created a feeling of security and collaboration (see Q14). This coach should guide them on diet, activity, behaviour, motivation and more. The participants wanted to be assigned one coach for the whole period and receive follow-up and coaching both in the app (e.g., video calls or chat) and in groups with other users. Group follow-up should only include people with T2D who are in the same situation. To most, coaching and follow-up was said to be necessary in the beginning. This could later be reduced when new habits have been adopted (e.g., replaced by a chatbot). Conversely, some said coaching can be frightening in the beginning and preferred a more gradual approach.

*“There’s something about having that one coach who has knowledge about diabetes.” Q14 (Female, 50-59 years, T2D)*

Importantly, the coach should alert the user if critical events occur (also confirmed by one user in the questionnaire), to create a feeling of safety for the user. The participants want follow-up when things are not going according to plan, not when things go smoothly. The coaching sessions can take 10–15 minutes or longer, depending on the situation and needs of the user.

Stigma and feelings of shame associated with the disease is common (see Q15), and participants miss communicating with people in similar situations (i.e., other T2D patients). Therefore, participants wanted a functionality that allows for contact with peers, friends, or family members, similar to a Facebook group where only invited people are allowed in. Such groups can increase motivation and feeling of support but should ensure privacy and security.

*“Many do not want to say that they have diabetes. Would preferably keep it a secret.” Q15 (Male, 60-69 years, T2D)*

The questionnaire showed that ten were positive to communicating with a personal coach through the app (one answered no, whereas the remaining two were unsure whether it could be fussy). Everyone wanted to share blood glucose data with their coach, twelve wanted to share medication use, ten wanted to share information on dietary intake, body weight, and physical activity, and seven wanted to share their mood or psychological state. Regarding frequency of follow-ups, most wanted monthly sessions.

#### Theme 4: Functionalities and content of the app as an interactive tool.

The participants wanted the app to include functionalities for blood glucose, diet, physical activity, body weight and other health parameters (sleep, heart rate, oxygen saturation, menstrual cycle, and electrocardiography). For all parameters, colour coded historical graphs were wanted, and these were considered important for motivation and learning. Some pointed out that the e-health program should not drain the smartphone battery more than other apps.

Participants highlighted the importance of including blood glucose data in the e-health program (see Q16) and wanted to see this in context with other factors such as diet, physical activity, sleep and more, preferably via graphs, to see how they affect blood glucose levels, and conversely. However, some participants claimed to have knowledge about what affects their blood glucose and would not need this functionality. If using a CGM device or traditional blood glucose meter, such data should be automatically transferred to the app.

*“I want that, when I measure, those data can go to the app. Because it’s difficult today, I insert the data manually on the phone when I measure my blood sugar.” Q16 (Male, 60-69 years, T2D)*

Some participants already used, or had previously used, a CGM, whereas others had never heard about it but were interested to learn more. Moreover, CGMs were perceived as motivating, easy-to-use, and more discrete and comfortable compared to conventional blood glucose meters but were said to be too expensive (see Q17). Others would invest in such a device as they thought it would benefit them in their self-management. Several participants stated that CGMs should be provided by health authorities, and felt their disease was underestimated or discriminated compared to type 1 diabetes (T1D) where patients receive such devices free of cost. This underpinned the belief that T2D is a less “serious” lifestyle disease that should be manageable if only the patient would make better lifestyle choices. CGMs can improve learning and give overview and a sense of control, but some claimed it could be stressful and wanted to focus more on their HbA1c instead of checking their blood glucose levels several times each day.

*“I must say that it’s very motivating. Instead of pricking your fingers […]. It’s a shame that it has to be so incredibly expensive.” Q17 (Male, 70-79 years, T2D)*

For diet, an overview of healthy options or alternatives should be provided (see Q18), and the wish for a swap list was brought up several times. This list should compare different foods and recommend the best alternative for the user (e.g., compare two types of bread and recommend the one that will cause the smallest glucose spike). It should make food shopping easier for the user and at the same time be educational (see Q19). Furthermore, participants wanted to see nutritional content of the different types of food, including carbohydrates, protein, fat, fibre, vitamins, and number of calories. Several participants wanted a scanning functionality, which provides this information by scanning the bar code of the chosen food, as they thought this would help to make better choices during shopping. However, some claimed such a functionality could be stressful and they would not use it. Others suggested options to photograph the meals and receive feedback from their coach based on those photographs. Although caloric information was useful, participants did not want to count or register this themselves. When registering foods, feedback from a coach was wanted (e.g., is this healthy or unhealthy, and what it means).

*“Give us some alternatives, give us some thoughts on how we can think differently.” Q18 (Female, 50-59 years, T2D)*

*“But if it had said “I recommend you use this, instead of butter I recommend mayonnaise” or something like that […] then I would go buy that and use that. I would.” Q19 (Male, 60-69 years, T2D)*

Registration of food and drink was considered essential and should be as simple as possible. The participants wanted personalized diet plans that could be adjusted to cater for the whole family if needed. However, the diet plans should not be too rigid, i.e., it should include weekly menus with several alternatives. Moreover, the meals should be varied and healthy, and include common ingredients used in Norway.

For the participants it was important to be able to track both body weight and physical activity. Physical activity data should be automatically transferred from devices (e.g., smartwatches), whereas body weight may be inserted manually or automatically from a smart scale. Goal setting was important, and participants wanted rewards or points when achieving them (see Q20). Activity plans should be personalized, and the app should include videos on how to perform exercises and how to train if injured. Guidance during activity (e.g., when to take breaks, increase pace) was wanted and were considered motivating. Different types of training ought to be included and recommended (e.g., both resistance and endurance training). Weight reduction was especially important to many participants with T2D, and functionalities for this should be enjoyable and motivating.

*“You receive a message that you achieved this and this goal, then I receive a thumbs up or a trophy or something like that, and I think that is very inspirational.” Q20 (Male, 60-69 years, T2D)*

The app should provide reinforcement/positive feedback (e.g., fireworks and affirmations) and give reminders for diabetes-related issues (e.g., checking vision and feet, seeing their doctor, and taking medication). Most importantly, the e-health program should provide an all-in-one solution, meaning that the users can have all relevant information in one place, as opposed to using different apps for different purposes (one app to track diet, another one for blood glucose measurements etc.) (see Q21).

*“Get everything in one place. As it is today, you quickly need four-five-six apps, and that’s just too much.” Q21 (Male, 60-69 years, T2D)*

The questionnaire confirmed that, out of 13 participants, all wanted to register their blood glucose and diet, ten wanted to register their body weight and physical activity, seven wanted to register sleep, whereas two wanted to register their mood or psychological state.

#### Theme 5: Tailoring.

Participants expressed a desire to tailor their user experience to their own preferences while using the e-health program. This included tailoring preferred functionalities such as caloric information and content of foods, types of health data registration like sleep, heart rate etc., type of physical activity to engage in, menstrual cycle, goal setting, and a scanning functionality for foodstuffs (see Q22). Furthermore, frequency and type of notification and reminders, preferred method (video vs. chat) and frequency of follow-up and coaching, should be tailored to the individual needs. They also wanted the option to sign up for educational courses depending on individual needs and interests, to prevent being overwhelmed. More detailed information can be included in courses the users sign up for by choice.

*“That’s maybe what’s most important, that it’s tailored, that it’s not made for people who are very concerned about health […], that group probably has reasonably good health already. So, it has to be made for us, or for me.” Q22 (Male, 50-59 years, T2D)*

### Suggested paper-prototypes from the final focus group meetings

The paper-prototypes provided by participants in the second (final) focus group meetings visualized their preferences and suggestions for the mobile application in the proposed e-health program. To limit time spent on the task, only the following screens/pages were included in the session: home screen, blood glucose page, and diet and activity registration pages. However, an empty screen was provided and could be filled out if time. All participants provided their individual prototypes, resulting in 16 unique contributions.

**Fig 1:**
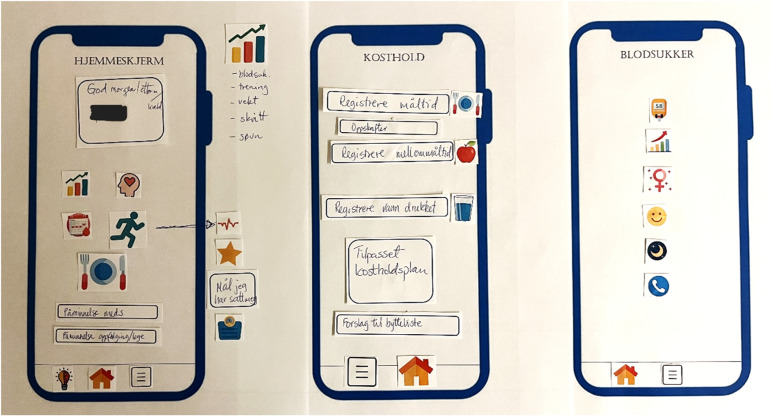
Paper-prototype from one of the participants, suggesting functionalities for diet, physical activity, blood glucose, education, mood, sleep, historical graphs for different parameters, and reminders. Hjemmeskjerm =  home screen, Kosthold =  diet, Blodsukker =  blood glucose.

**Fig 2:**
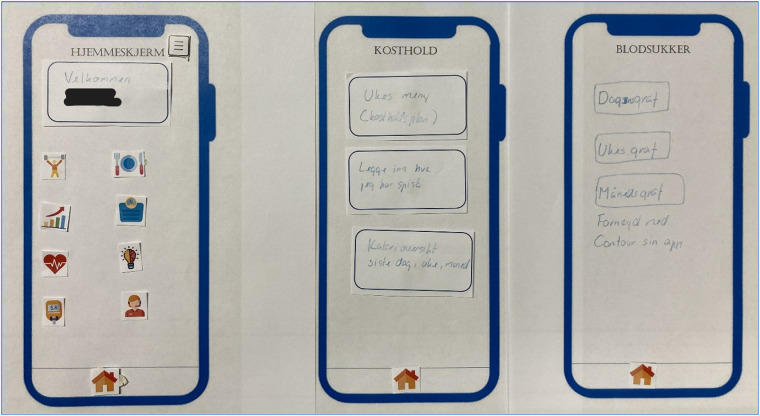
Paper-prototype including important functionalities, a diet registration and overview screen, and a blood glucose screen in which the participant claims to be satisfied with the CONTOUR Diabetes app [31] interface.

Fig 1 depicts one participant’s preferred user interface of the e-health program, including several functionalities, shortcuts, and buttons. Furthermore, Fig 2 shows a paper-prototype including the same personal greeting in the home screen as seen in Fig 1, but with slightly less information, and a simpler interface for the diet screen.

**Fig 3:**
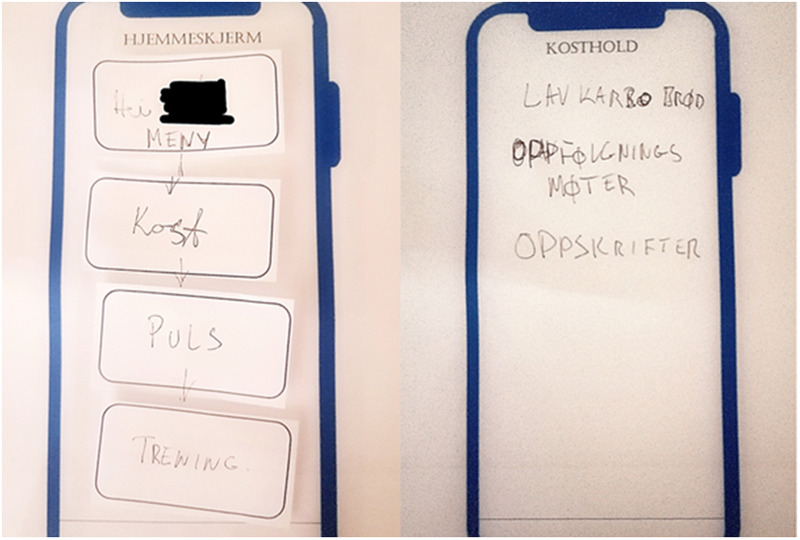
Paper-prototype with a personal greeting and descriptive text boxes for diet, heart rate, and physical activity in the home screen, and information about dietary components, including recipes, and a follow-up functionality.

From the prototyping sessions it was clear that the differences in needs and preferences between participants were large, further confirming the importance of tailoring opportunities to allow users to adjust the e-health program to make it “their own”, and thereby facilitate motivation and long-term usage. Fig 3 exemplifies this point by showing a paper-prototype containing far less information and including more descriptive text boxes, which may contribute to better understanding and clarity for some, compared to the use of icons to represent different functionalities.

### Questionnaire results

Written answers from the questionnaire are listed in Table 1 below, and explores expectations to the e-health program, importance of follow-up, and how sharing information with a coach can impact self-management of T2D. Other findings from the questionnaire have been provided earlier in this paper.

**Table 1:** Written answers to the three questions in the top row, provided by 13 participants.

Participant	Question 1	Question 2	Question 3
	**What do you think an e-health program must include for you to use it in the long-term?**	**Is follow-up with a coach through the e-health program important for you?**	**How can sharing of information with your coach impact self-management of your disease?**
P1	*Easy to register, easy to use, good overview of diet/blood sugar/training/coaching.*	*Yes, it is important for my focus on best possible health.*	*Positively, I think, because the coach will have an overall picture of how I’m coping.*
P2	*App and blood glucose meter can communicate. App and smartwatch communicate.*	*Yes, think it’s important.*	*That I become confident that the efforts I make are right.*
P3	*Motivational.*	*No, not really.*	*Have no idea.*
P4	*All necessary information in one picture with hyperlink to go in and see progress.*	*Can be of interest.*	*Can give access in the app.*
P5	*Easy to use, easy to register, motivation, clear, follow-up.*	*Yes.*	*New medications and new and better equipment can come. Development and research happen quickly, always something new coming.*
P6	*The tool must be easy to use and understand. Registration of nutrients must be easy and easy to find composition. Results must be presented so they motivate to continued usage.*	*There must be reminders for follow-up according to treatment.*	A *good overview of my disease can give the coach ideas about other treatments than what is today’s treatment.*
P7	*Colours, easy to use, accordance with each other, motivational to use, follow-up.*	*Yes.*	*Will motivate me.*
P8	*Clear, easy to register things, and motivational.*	*Feedback and tips are important.*	*I feel that I have great self-management today.*
P9	*Simplicity (easy to use for all age groups), good overview, easy to send reports to the doctor.*	*Basically not. Can be too fussy. But if it could be possible to contact a diabetes nurse that would be good.*	*It can be nice to get confirmation that the things you do are right. Often you feel “alone” trying to figure out all about having diabetes. Sharing of information with a coach can help, I think.*
P10	*Easy to use and show progress.*	*Can be positive.*	*Uncertain.*
P11	*Everything gathered in one app, easy to use that is motivating and can give learning.*	*Yes.*	*Hopefully get control of weight and diabetes.*
P12	*Easy to use. Easy to register things.*	*Follow-up conversations, feedback.*	*Important for motivation.*
P13	*Easy to connect to blood glucose meter, watch etc. Overview of various measurements.*	*Like having someone paying attention.*	

P =  participant

## Discussion

This qualitative user preference study aimed to inform the development of a comprehensive e-health program for T2D, by investigating end-user preferences and suggestions for implementing a successful e-health solution that promotes long-term usage.

Based on the literature and field experience, it is evident that there is a gap between what end-users want from e- and m-health tools, and what is currently available on the market. In particular, tailored e-health programs or apps targeting the T2D population and their individual needs, was confirmed by end-users in this study to be lacking. Hence, we believe that our planned e-health program could help fill this gap and provide a comprehensive tool for people with T2D, based on results in this study.

### Motivation – an essential factor for long-term usage

Our participants highlighted the importance of an all-in-one solution to reduce the burden of needing several apps to monitor and follow up on their health, which should include integration with devices such as smartwatches and blood glucose measuring tools (CGMs and/or traditional blood glucose meters) to facilitate ease-of-use and thereby long-term usage. Tracking progress over time using color-coded historical graphs was essential for motivation. Generally, a clean and simple interface, including simple data entry methods, may increase the users’ satisfaction with the tool [27], and our participants expressed that the e-health program must be easy to use and understandable in order for them to use it.

Consistent with our findings, studies show that reinforcement (through reminders and rewards) [32] and automatic data collection from devices are important features that can increase motivation and usage [27,33]. Automatic data collection does not require the user to input information or interact with the app in other ways than simply opening it and viewing the data. This is possible for physical activity and CGM data, when there can be established a wireless connection between the device and the app [33]. However, registration of food intake requires some manual interaction. Automatic data collection was particularly important for the participants in our study, especially for physical activity parameters and glucose data.

Compared to the general population, people with T2D are less likely to use health apps, often due to lack of awareness of the existence of such apps [34,35], whereas people with T1D use them more frequently [35]. This was also brought up in our focus groups, and some participants were unsure about which apps to use, and how to choose between the large number of existing apps on the market. Furthermore, many apps are aimed at improving health in general, and not mainly focused on T2D. This point was made in the focus groups, where participants expressed a concern for not finding health apps targeting T2D. To motivate end-users with T2D to engage in technological tools, they should be developed for the purpose of improving diabetes-related health outcomes. Studies have also shown that usability-related dissatisfaction with diabetes apps is another reason for low usage in the T2D population [36,37].

Although their study included participants with T1D, Cafazzo et al. [38] reported that end-users want apps that are quick to use, discrete to use in public, provide rewards, and provide safe information sharing with caregivers. These topics were also discussed in our focus groups, where rewards and a program that is quick to use were highlighted as especially important.

### Increased knowledge for better self-management

Living with pre-diabetes and T2D was reported as challenging for many participants, especially due to lack of knowledge and education about diabetes. To enhance learning, our participants wanted to be provided with educational content to understand their disease, and what affects it. This included dietary concerns, physical activity, physiological parameters such as heart rate, blood pressure, sleep, and menstrual cycle, and psychological factors (e.g., mood).

Education has been shown to increase diabetes self-management [39,40], and may therefore ease diabetes distress and potential subsequent negative health outcomes. Use of glucose sensors (CGMs) in patients with T2D can give greater improvements in glycaemic outcomes compared to standard fingerstick monitoring [41]. In our study, several participants felt discriminated by health care personnel (HCP) for not being offered such technology even though it could aid their self-management and learning, and thereby decrease their diabetes distress and improve overall health.

Similar to our study, Bradway et al. [14] explored end-users’ needs and expectations for an m-health patient-gathered data sharing system, and included patients with T1D, T2D and HCP. Their participants claimed they did not receive enough information about their disease, and T2D patients were unsure of reasons for fluctuations in their blood glucose levels. This confirms the need for education and increased knowledge, found in our study.

### Follow-up and support – an important aspect of diabetes management

Follow-up is crucial in diabetes management and should be offered through the program, where each user is assigned a personal coach whom they may interact with via chat, video, or audio. Social groups with peers, family, and friends were highlighted, and may reduce the burden of T2D for the user by allowing them to share experiences and receive support.

Patient-gathered data can be especially useful in meetings between patients and HCP and is an important aspect of disease management. However, it is unclear how this can be done to effectively aid both patients and HCP in a sustainable way. In the beforementioned study by Bradway et al. [14], they found that a clear overview of relevant data, shared decision-making and mutual understanding between patient and HCP were important in consultations. Their study confirmed that such interaction allowed both parties to explore the data and collaborate in a better way.

As many e-health and m-health tools are stand-alone systems used by the patient (and not HCP), Ruissen et al. [42] developed a clinical-based support system to enhance self-management and shared decision-making between diabetes patients (both T1D and T2D) and HCP. They found that it improved glycaemic control, self-management, and quality of life, without increasing hypoglycaemia. Their e-health program included manual and automatic data collection, overview, and feedback, consistent with the aims of our e-health program. However, they used the program during consultations with HCP, and provided targeted interventions, psychological exercises, and psychoeducation if the users experienced barriers to self-management.

A systematic review by Greenwood et al. [22] showed that studies including two-way communication between both patient and HCP improved several health outcomes. Furthermore, they showed that improvements in HbA1c were greater when using mobile communication compared to web-based interventions. Hence, the inclusion of two-way communication in our e-health program will be of great importance and should ideally be extended to HCP in GP offices and treatment facilities beyond the use of coaches.

### An interactive app as part of the e-health program

A recent study by Pienkowska et al. [43] included eight T2D patients to co-design a diabetes education app prototype. Although their prototype was already developed prior to the co-design and evaluation, and most participants stated that they had more than average knowledge about T2D, the researchers explored similar topics and themes as in our study. Notifications (regarding education) were said to be acceptable if not received too often, which supports our finding and calls for the important possibility for tailoring of such content. Pienkowska et al. also highlighted the importance of tailoring/personalization, and inclusion of evidence-based and up-to-date information. However, their app did not offer remote consultation, which is an important part of our planned e-health program. Also, they included only eight participants who stated to be at least above average knowledgeable in T2D, and all participants used and were very satisfied with apps. Our study included 16 participants in which many claimed to know little about T2D. Still, many similar results were found. Since Pienkowska’s study was performed in Singapore, it can be assumed that cultural differences play a role, both in the needs and preferences of participants, but also on the level of knowledge about the disease and what affects it. Furthermore, they were recruited from online diabetes groups, and this may explain the high level of knowledge in the sample (i.e., high interest and engagement in diabetes-related matters).

Boyle et al. [35] explored use of, and beliefs about, mobile apps for diabetes. Among app-users with T2D, functionalities for blood glucose were considered the most important ones (e.g., blood glucose diary, reminders to check glucose levels). Their study showed that functionalities for a calculation device for insulin doses, diary for meals and carbohydrate intake, reminders to check blood glucose, and contact details for their diabetes team were wanted features in future diabetes apps. This is in line with our findings, except the insulin functionality, which was not discussed in our focus groups since our e-health program primarily targets lifestyle intervention in people with prediabetes and T2D not using insulin.

### One size does not fit all

Despite the promising potential of e-health and m-health tools for improved health outcomes, such tools are subject to low adherence and high drop-out rates [44,45]. Hence, designing tools that facilitate motivation and thereby long-term usage, is crucial. Tailoring and or personalization increases the likelihood of adherence, long-term usage [46] and the user’s interaction with the tool [47], which may consequently also increase the effectiveness of e-health/m-health interventions [32,46]. Tailoring is essential to personalize the program to the individual end-user and this feature was highlighted as very important by our participants. Their suggested paper-prototypes and the answers to the questionnaire confirmed the needs and preferences discussed in the focus group meetings.

### Implications for future development

In the study by Bradway et al. [14], HCP participants expressed concerns regarding resource management if implementing a patient-gathered data sharing system, because it would require training and support. It is critical that such tools are developed so that they are easy to use for all parties: That they can reduce the workload for HCP and create enhanced outcomes compared to traditional treatment for patients.

In our focus group meetings, participants believed using the e-health program could improve consultations with their GPs. For instance, reviewing outcomes in collaboration with their GPs could contribute to mutual understanding and provide a clear overview of what the patients had done to improve their health, as well as identifying existing challenges and barriers.

An important aim of our e-health program is to reduce the workload for HCP (e.g., GPs) by relocating tasks to coaches through the program. Therefore, future research should investigate how such an e-health program could aid HCP in T2D treatment and ways to implement such a tool without increasing the workload for the HCP.

### Strengths and limitations

This study allowed sixteen people from both rural and urban populations in Norway to participate and voice their opinion on the planned e-health program. Including participants from all over the country is especially important in a country like Norway, where the population is spread across a large geographical area. A great number of T2D patients live far away from healthcare services like GP offices, hospitals or other treatment facilities. It is especially important to include this latter group, as they may benefit from the use of e-health tools to reduce travel distance and costs and thereby receiving healthcare services similar to those living in urban areas.

The study sample included 75% males, which is contrary to many research studies, where females make up the largest proportion of the sample. However, as this study focused on informing the development of a *technological tool*, perhaps males were more likely to participate due to higher interest in technology or feeling more technology proficient, compared to females. As perspectives from both technologically proficient and non-proficient participants were wanted in this study, the recruitment process could have emphasized this even further, potentially increasing the number of people not used to apps and similar tools to attend. Nevertheless, our study included a balanced combination of proficient and non-proficient participants.

Moreover, recruitment took place mainly online, requiring potential participants to have access to a computer or smartphone. Since the T2D population consists of many middle aged and elderly patients, it can be assumed that many were not able to participate due to little or no use of computers/smartphones and social media apps.

### Recommendations

Based on findings from this study, a list of recommendations was created (Table 2). The list contains the most crucial functionalities and features to include when developing e-health and/or m-health tools for people with pre-diabetes and T2D.

**Table 2:** Recommended features to include in e-health/m-health tools for T2D.

Component	Recommendations
Interface	• Clean, simple, understandable, and easy to use • Little to no manual input • Balanced use of colours (adjusted to suit people with reduced vision etc.) • Historical, colour-coded graphs, visualizing progress
Data transfer and connectivity	• Automatic transfer from diabetes and activity devices (e.g., CGMs, glucose meters, smartwatches) • All-in-one solution
Registration of health parameters	• Including blood glucose, diet, physical activity, body weight, psychological state/mood, sleep, menstrual cycle, oxygen saturation, and other relevant health parameters • Swap list and scanning functionality for managing diet/nutrition
Communication and follow-up	• Chat, audio, and video calls with trained coaches or HCP • Long-term follow-up with assigned coaches
Diabetes education	• Educational courses (interactive webinars, quizzes, lectures) by trained staff in diabetes, nutrition, physical activity and more • Access to a trustworthy source of diabetes-related information
Tailoring of functionalities and features	• Adjustable: information and content of foods (e.g., calories), types of health data registration, type of physical activity to engage in, menstrual cycle, goal setting, and a scanning functionality for food • Frequency of notifications, reminders, and follow-upsType of notifications and reminders • Preferred follow-up method (chat vs. video) • Sign-up-functionality for educational courses

## Conclusion

This user preference study explored preferences and needs for a new, comprehensive e-health program for T2D. Lack of necessary diabetes knowledge was common among participants, and education was said to be essential in improving self-management. Essential desired functionalities included registration and overview of several health parameters, long-term follow-up and coaching through communication platforms within the program, automatic data transfer from different devices, and educational courses. To ensure end-users’ satisfaction and increased motivation to use the e-health program in the long-term, tailoring of desired functionalities and content was said to be crucial.

Future research should include HCP to explore how an e-health program can be used during consultations, and explore what requirements and functionalities are desired by HCP, while simultaneously saving resources.

The list of recommendations presented in this paper (Table 2) was based on feedback from end-users and provides a checklist for future development of e-health and m-health tools for pre-diabetes and T2D.

## Supporting information

S1 Appendix Recruitment flyer. (PNG)

S2 Appendix Consent form. (DOCX)

S3 Appendix Interview guide 1. (DOCX)

S4 Appendix Interview guide 2. (DOCX)

S5 Appendix Questionnaire. (DOCX)
