# Supplementary figures and images for: What are end-users’ needs and preferences for a comprehensive e-health program for type 2 diabetes? – A qualitative user preference study
Source: PLoS One. 2025 Mar 3;20(3):e0318876. doi: 10.1371/journal.pone.0318876 (PMC11875348; doi:10.1371/journal.pone.0318876)

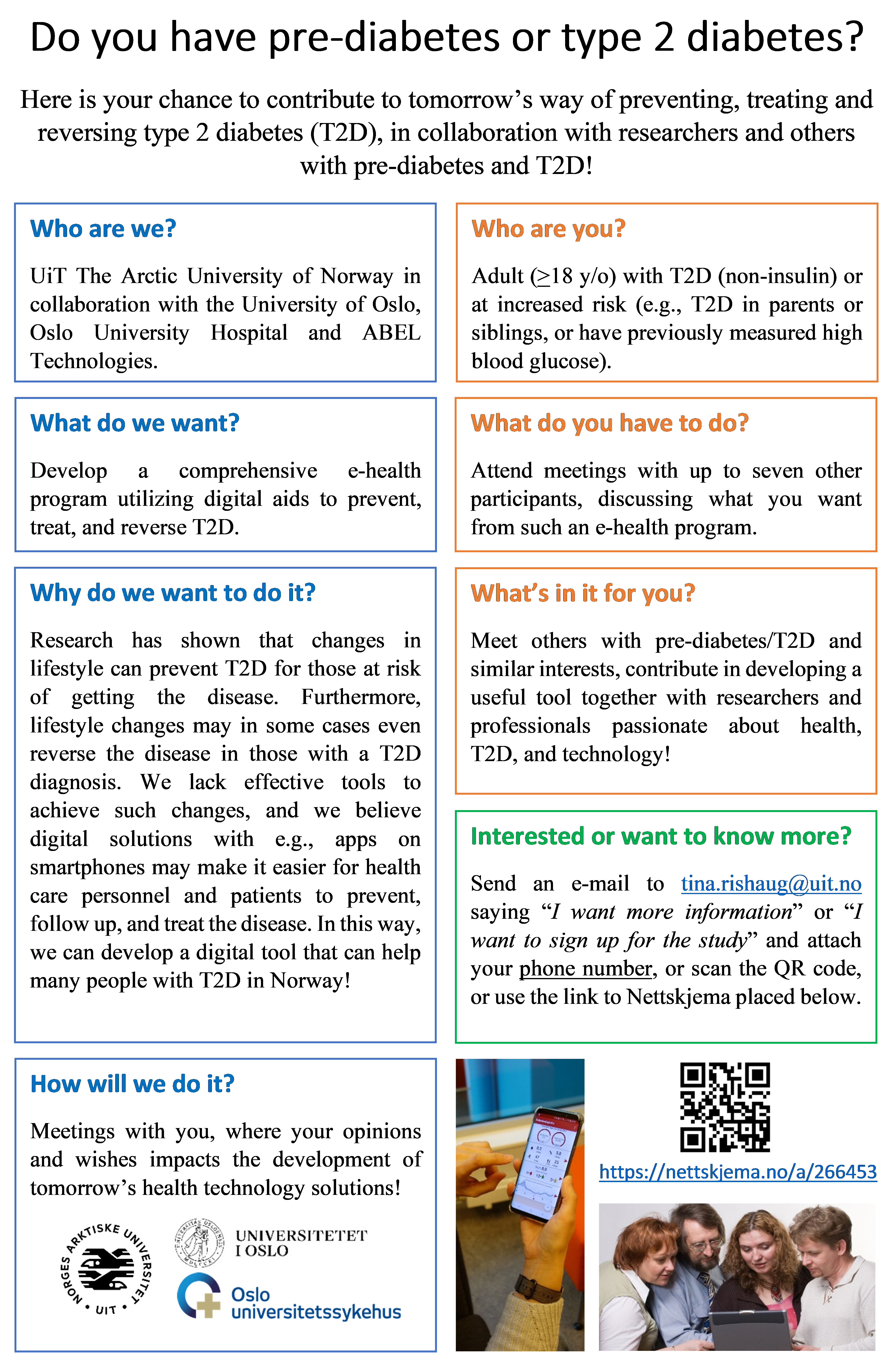

Supplement: S1 Appendix — (PNG) [file pone.0318876.s001.png]
