# Supplementary material for: What are end-users’ needs and preferences for a comprehensive e-health program for type 2 diabetes? – A qualitative user preference study
Source: PLoS One. 2025 Mar 3;20(3):e0318876. doi: 10.1371/journal.pone.0318876 (PMC11875348; doi:10.1371/journal.pone.0318876)
Supplement: S2 Appendix — (DOCX) [file pone.0318876.s002.docx]

**Do you want to participate in the research project “DiaMestring – pre-study”?**

# This is a question for you to participate in a research project with the aim of gathering relevant information, preferences and needs your as an end-user may have in the use of a comprehensive e-health program for prevention, self-management, and remission of type 2 diabetes (T2D). In this form we will provide information regarding the aims of the project and what participation will mean for you.

**Aim**

The aim of the project is to conduct physical and digital focus group meetings with participants who have either pre-diabetes or T2D. During these meetings, you will contribute with information, preferences, and needs on what functionalities you believe an e-health program should contain. This e-health program is under development, and you will have the opportunity to contribute to the design and development.

You will be asked to attend two focus group meetings, where each meeting will include up to eight participants in total, and last approximately 120 minutes (including a 15-minute break).We will focus on discussions and conversations about functionality and similar that can stimulate to the use of an e-health program, motivation and self-management, and how an e-health program may prevent, treat, and lead to remission of T2D in the best possible way. You will participate in in so-called paper-prototyping. A draft of the e-health program is presented (on paper), and you will have the opportunity to voice suggestions and wishes based on your own experience with the program, and what you require from such an e-health program for it to be as effective as possible. For those who participate digitally we will use a digital solution for prototyping (Miro). Audio recording will be used in both physical and digital meetings.

This pre-study is a research project with the aim of developing a comprehensive e-health program which is planned used in a large, nationwide randomized controlled trial (RCT).

**Who is responsible for the research project?**

UiT The Arctic University of Norway (UiT), The University of Oslo (UiO), and Oslo University Hospital (OUS) are responsible for the project.

The technology company ABEL Technologies is a partner in this pre-study and are responsible for developing the technology in collaboration with the project team mentioned above.

**Why are you asked to participate?**

The recruitment process takes place on social media directed by the Norwegian Diabetes Association, as well as through our collaborating partner at OUS and The University Hospital of North Norway (UNN). Adults (>18 years) with T2D or elevated blood glucose values/increased risk of developing T2D are welcome to participate. You have no conditions making it difficult to eat a normal diet or be normal physically active (e.g., serious heart disease or lung disease), or making it difficult to use a mobile phone (e.g., reduced vision). You are willing to attend focus group meeting with other, either physically or digitally, and consent to audio recordings during the meetings.

# What does it mean for you to participate?

If you participate in this pre-study, you will attend focus group meetings where we will use audio recording and a questionnaire to gather information. Each focus group meeting will last approximately 120 minutes, and you will be asked to attend up to three such meetings (over the course of four weeks), where up to seven other participants are present.

Prior to the first meeting, we will ask you for information about age, gender, and education level. This information will be used to describe the study population and will not be possible to trace back to you.

You may be asked to participate in testing of the e-health program when it has been developed. We wish for everyone who has participated in this pre-study to participate in the testing, but this is completely voluntary.

**Participation is voluntary**participation in this pre-study is voluntary. If you choose to participate you may withdraw your consent at any time without providing a reason. All your personal information will then be deleted. If you do not wish to participate, or choose to withdraw at a later stage, there will be no negative consequences for you.

**Your privacy – how we store and use your information**

Information about you will only be used for the purposes we have informed you about in this form. We handle your information confidentially and in accordance with privacy policies.

- Project managers at UiT and UiO will have access to retrieved data.
- Whereby will be used to conduct meetings during digital focus group meetings, where no data will be stored.
- Your name and your contact details will be replaced with a participation number which will be stored in a separate name list, stored separately from the other data. Retrieved information from audio recordings will be safely saved behind to-factor authentication by the managers at UiT and stored on encrypted memory sticks locked in a cabinet.
- Transcription of audio recordings will be conducted on an off-line computer for additional security, where only your participation number will be used. Audio recordings will be deleted after transcription.
- Results from the focus group meetings will be shared with ABEL Technologies (no contact or personal information).

Transcription of audio recordings will be performed by project managers at UiT on university owned equipment.

Upon publication, no personal or contact information will be shared, and no participants should be recognizable. Only information about wishes and needs from participants will be shared in a publication.

**What happens to your personal information when the project ends?**

The project will, according to the plan, end during the spring 2023. After the project ends, all data material with your personal information will be deleted.

Audio recordings from focus group meetings will be archived and used in the development of the e-health program. In the planned main study (after this pre-study), the wishes, needs, requirements etc. arising from the focus group meetings, will be used to justify the design and functionalities of the e-health program. This information will be stored on encrypted memory sticks in a locked cabinet at UiT, where only research managers have access. Data material will be stored until the main project is finished, planned at the end of 2024.

**What gives us the right to handle personal information about you?**

We handle your personal information based on your consent.

Privacy Services (*Personverntjenester*) has assessed the handling of personal information by UiT and UiO in this project to be in accordance with current privacy policies.

**Your rights**

As long as you are identifiable in the data material, you have the right to:

- Insight into which information vi handle about you, and to receive a copy of this information.
- To correct information about you that is wrong or misleading.
- To have information about you deleted.
- To send a complaint to The Norwegian Data Protection Authority about the handling of your personal information.

If you have questions about the project or want to know more about, or make use of your rights, please contact:

- UiT The Arctic University of Norway, Eirik Årsand, e-mail: [eirik.arsand@uit.no](mailto:eirik.arsand@uit.no)
- The University of Oslo, Anne-Marie Aas, e-mail: [a.m.aas@medisin.uio.no](mailto:a.m.aas@medisin.uio.no)
- Our Data Protection Officer, Joakim Bakkevold, e-mail: [personvernombud@uit.no](mailto:personvernombud@uit.no)

If you have any questions regarding Privacy Services’ assessment of this project, please contact:

- Privacy Services (*Personverntjenester*) on e-mail ([personverntjenester@sikt.no](mailto:personverntjenester@sikt.no)) or phone: 53 21 15 00.

With kind regards

Eirik Årsand (professor, UiT) and

Tina Rishaug (researcher, UiT)

-------------------------------------------------------------------------------------------------------------------------

**Declaration of Consent**

I have received and understood the information about the project “DiaMestring – pre-study” and have been given the opportunity to ask questions. I consent to:

- Participation in focus group meetings.
- That conversations I participate in are recorded.
- That information I provide can be used until the end of the main study.

I consent to my information being handled until the end of the project.

----------------------------------------------------------------------------------------------------------------

(Signed by project participant, date)
