# Supplementary material for: What are end-users’ needs and preferences for a comprehensive e-health program for type 2 diabetes? – A qualitative user preference study
Source: PLoS One. 2025 Mar 3;20(3):e0318876. doi: 10.1371/journal.pone.0318876 (PMC11875348; doi:10.1371/journal.pone.0318876)
Supplement: S3 Appendix — (DOCX) [file pone.0318876.s003.docx]

**Semi-structured interview guide**

DiaMestring – pre-study

| **FIRST MEETING**  **INTRODUCTION (no recording)** | | |
| --- | --- | --- |
| **Goal** | **Activity** | **Instructions** |
|  | Welcome  Ice-breaker activity to get to know each other | **Welcome**   - Anne-Marie welcomes everyone - Show PowerPoint   *Digital: turn on your camera* 😊  **Introductions**   - Leaders introduce themselves and their background. - Participants introduce themselves. - Who you are (name, age), where you’re from (if you want to share) - Everyone receives name tag and participation number - **Reminder:** we do not record this part. - Researchers describe their role in the project and meeting, suggestions: - Meeting leader (TR) - Researcher (EÅ) - Researcher (AM) - Towards the end we will look at CGMs!   **Ice breaker:**   1. What is your dream destination? 2. What is the best advice you ever got? 3. What skill/talent would you like to have if you could choose? 4. If you could interview anyone in history, who would you choose?   *Participants are given 2 min. each to answer the questions. They may skip questions if wanted.*  *Digital: everyone is given some minutes to write down answers beforehand, then we will go through them together.*  **Experiences with pre-diabetes/T2D**   - Your experience with T2D or pre-diabetes (if you want to share) |
| Give participants an overview of the day and the meeting topic | Meeting plan and goals | **Overview of the meeting and topics**   - Approx. 120 min. discussion including 15 minutes break. - Everyone contributes with what they want in the discussion, we wish for everyone to be able to share things. - The goal is to explore what you want from an e-health program. |
| Establish how everyone should behave and what is expected from the participants and meeting leaders | Meeting rules | **Rules**   - Everyone’s opinion and thoughts are important, there is no wrong opinion, and no right conclusion to the topics. - We ask you to focus on your role and your wishes as a participant – try not to assume what others mean or think. - Everyone should feel safe and comfortable. - We are all different with different experiences. - Respect other people’s opinion and what they share. - You may disagree, but be polite. - Ask in a nice way if there are things you don’t understand. - We encourage you to stick to the topic, and we will give a reminder if you go off topic. - We will record part 2 of this meeting, and the next meeting. - We will not use sensitive or personal information. If you wish to share something but do not want it to appear in the transcript, please let us know, and we will take this into account. - We will not discuss medical treatment or similar in this meeting, please contact your doctor if you wish to discuss such matters. - Nothing that emerges from this meeting should be used in personal treatment but should be discussed with your doctor. - We need feedback from EVERYONE, especially those of you who have less experience with use of e-health tools, and who may not be as invested, or have a lot of knowledge about technology. All feedback is valuable to us! |
| **5-MINUTE BREAK** | | |
| **PART 2 – DISCUSSION (with recording)** | | |
|  |  | Start recording (inform about this!)  **Topic of the day**   - Your experience living with pre-diabetes or T2D. - Your knowledge or, or experience with, e-health programs or apps. What you think of them, are there things to be improved etc.   **Explanation**   - We ask questions that you answer and discuss as a group. - We will write down important points to discuss closer together later. |
| Mapping challenges the participants have related to their disease/pre-diagnosis to tailor the e-health program accordingly | Discussion 1:  Health challenges related to your disease | **Question 1:**   - What do you consider as the biggest challenge with having pre-diabetes or T2D? How do these challenges affect the following:   - Your general health   - Everyday life and work   - Family, friends and social life   - Psychological aspects (everything from mood to more serious things)   - Other?   - How do these things affect your view of body weight, body image, physical activity and similar?   **Does anyone have anything else to add?** |
| Clarify experiences the participants have, what thoughts they have about e-health, suggestions for improvements etc. | Discussion 2:  Use of/experience with e-health or similar apps/programs | **Question 1:**   - What is your experience with e-health tools? E.g., an app or internet-based program.   *Not everyone has used such tools before, and that is completely fine!*  **Question 2:**   - Do you use any such tools today? - What is positive? - What is negative? - Are they valuable/useful to you?   - Why/why not?   - What would it take for them to be valuable/useful to you? - Why do you not use such tools?   *Everyone answers in turn. You may skip a question if you do not wish to answer.*  **Does anyone have anything else to add?** |
|  | Discussion 3:  Diet and use of e-health tools | Diet and e-health tools.  **Question 1:**   - Is it important for you to have an ovewview of your diet? E.g., calorie content, carb content. - Why/why not? - Do you use any form of registration of your intake of food/drinks? - Would you like a personalized diet plan?   **Question 2:**   - What do you wish to have an overview of? E.g., nutritional content of dishes or in each foodstuff, calorie needs etc.   - Can this take too much time/become too complicated?   - How often do you think you would have done this:     - Every day     - Once a week     - Once a month     - Only prior to doctor’s appointements     **Question 3:**   - Do you think such information contributes to you changing your diet for the better? E.g., eating more healthy, more regular, adjust amount and portions etc. |
| **10-MINUTE BREAK** | | |
|  | Discussion 4:  Body weight and physical activity and use of e-health tools | Body weight and physical activity and e-health tools.  **Question 1:**   - Would you like to register your body weight and activity level?   - Why/why not?   **Question 2:**   - Would a graphical overview with changes in your body weight and activity level over time be useful? - Why/why not? - Easier to adjust or maintain body weight and being more active? |
|  | Discussion 5:  Other health measurements and use of e-health tools  Use of CGM in type 2 diabetes | Other health measurements and e-health tools.  **Question 1:**   - Would you like to measure and have an overview of your blood sugar? - Why/why not?   **Question 2:**   - Would you like to measure and have an overview of your sleep? - Why/why not?   **Are there other things you would like to register?**   - E.g., mental health, menstruation etc.   *Describe both pros and cons of CGM. Eirik will demonstrate its use.*  **Question 3**:   - Thought about using a CGM? - Positive/negative? - Is it acceptable for you to pay for this yourself (for own use after the study)?   - Price: 599 kr for 14 days |
|  | Ending and summary | Close the meeting with a short summary.  Discussion between everyone:   - Important points - What is essential to include - Things that are less important   How was this day?  Do you have any feedback for us?  Something we could have done better?  **For next time**  Download a free app of your choice. The app should be about health, e.g., training, diet or similar. You choose which one you want, but try to think about positive things about it, what is negative, are there things you notice that are good or not so good? Think about these things, and write them down if you wish, then we can share these things in the next meeting.  **Next meeting**   - When and where? - How long? - Topics - Motivation and self-management by using e-health tools - Prototyping   Thank you for participating today! |
