# Supplementary material for: What are end-users’ needs and preferences for a comprehensive e-health program for type 2 diabetes? – A qualitative user preference study
Source: PLoS One. 2025 Mar 3;20(3):e0318876. doi: 10.1371/journal.pone.0318876 (PMC11875348; doi:10.1371/journal.pone.0318876)
Supplement: S4 Appendix — (DOCX) [file pone.0318876.s004.docx]

**Semi-structured interview guide**

DiaMestring – pre-study

| **SECOND MEETING – PART 1** | | |
| --- | --- | --- |
| **Goals** | **Activity** | **instructions** |
|  | Welcome  Icebreaker | **Welcome**   - Introduce ourselves again - Participants introduce themselves   - Everyone is given name tags   **Sum-up**   - Last meeting - What the project is about - Short summary of discussion topics   **Icebreaker**  Dilemmas   1. Be able to see 10 years or 150 years into the future? 2. Always buy 10 things you don’t need when shopping, or always forget the one things you were supposed to get? 3. Always arrive 10 minutes too late, or always arrive 20 minutes too early? |
| Give participants an overview of the day and topics | Meeting plan | **Meeting plan**   - Approx. 120 min. discussion including 15 minutes break - Everyone contributes with what they wish in the discussion, we want everyone to be able to share things - The goal is to explore what you want from an e-health program, and how you would design it - Questionnaire - Prototyping |
| Establish how everyone should behave and what is expected from the participants and meeting leaders | Meeting rules | **Rules**  Same as last meeting!   - Everyone’s opinion and thoughts are important, there is no wrong opinion, and no right conclusion to the topics. - We ask you to focus on your role and your wishes as a participant – try not to assume what others mean or think. - Everyone should feel safe and comfortable. - We are all different with different experiences. - Respect other people’s opinion and what they share. - You may disagree, but be polite. - Ask in a nice way if there are things you don’t understand. - We encourage you to stick to the topic, and we will give a reminder if you go off topic. - We will record this meeting, but if you want us to pause the recoding to share personal information, we will do so. - We will not record sensitive or personal information. If it should happen, all such information will be removed from the transcripts and then deleted. - We will not discuss medical treatment or similar in this meeting, please contact your doctor if you wish to discuss such matters. - Nothing that emerges from this meeting should be used in personal treatment but should be discussed with your doctor. |
|  | Present topic of the day | Start recording (inform about this!)  **Topic of the day**   - Motivation and self-management when using e-health tools - Comprehensive health solution - Prototyping |
| Explore today’s solutions and what the participants think about these; functionality, what is missing, what is ”too much”? Does it lead to self-management, learning, and motivation? | Discussion | **Question 1:**   - Can you think of something that gives you motivation and helps your self-management when using e-health tools or similar? - For diet - For body weight and activity - For other health measurements   **Question 2:**   - How can the use of an e-health program contribute to changes in follow-up/consultations with your doctor or other health care personnel?   - Positive/negative?   - Why?   **Question 3:**   - What do you think about the possibility of video consultations with a coach/health care personnel?   - Positive/negative?   - Why?   **Question 4:**   - If you receive follow-up with a coach, how do you wish to receive this?   - Individually?   - In groups?   - Mix of both?   **Question 5:**   - Imagine you are using the e-health program now: would you like to get reminders?   - About: blood sugar, food, activity, sleep, more?   - How often?   - Possibility to choose frequency and type of reminders?   *The idea is that the e-health program can be adjusted to your registrations, e.g., if your blood sugar has been high lately, the app can provide information about this for you to learn more. Or if you have been sedentary lately, you may learn more about the importance of activity etc..* |
| **15-MINUTE BREAK** | | |
|  | Paper-prototyping  Time use: approx. 20 min | Everyone fills out the questionnaire. Ask if something is unclear. Hand in or raise your hand when you are done.  Everyone will receive paper sheets with the prototypes, pens, and markers.  **Instructions**  You seen a reprensentation of the product on the paper sheets. We will explain the plan for the product.  Imagine you are using the product: what makes sense, what should be placed where, what is redundant etc. Are the icons too big/small etc.   - Draw with pens and markers - What could be on the left side, on the top or bottom of the screen etc.? - What should be placed in front, and more easily accessible in the app?   **Question prototyping**:   - What colours do you think are fitting to use?   - How many colours?   - Can it be too much/little colour?   **Other questions:**   - Find out why they are doing what they are doing, ask why this and that in the prototype, find out the logic for what they do.   *Hand in the sheets or raise your hand, and we will collect them. We will let you know when the time is up. Digital group will take pictures of their prototype and send to the group leader..* |
|  | Educational component | **Question 1:**   - Would you like to receive educational content about how diet, activity, and weight reduction impacts your T2D? - Why/why not? - How comprehensive? - How often? - Would you like the possibility to ask questions and/or discuss with other participants? - On which platform?   - App, video conference, physical …   - E-learning course with videos and quizzer for you to take when you feel like it   - Webinar   **Question 2:**   - Would you like to receive practical tips on how to implement this new knowledge into your everyday life to prevent or treat T2D?   **Question 3:**   - Would you like a personalized activity plan in the app? |
|  | Ending | **Closing question**   - If you could decide one thing about this e-health program, what would it be?   **Ending**   - How has this day been? - What do you think after these meetings? - What are your thoughts about the prototyping session? - Are there things we could have done better? - Other feedback?   Digital group: remember to take pictures of your prototypes and send to us!  Thank you all very much for participating in these meeting, and for your valuable information and feedback!  Info: everyone who has participated will be offered to participate in testing of the program when it has been developed. We will contact you via email, and you can decide whether you want to attend or not. |
