# Supplementary material for: What are end-users’ needs and preferences for a comprehensive e-health program for type 2 diabetes? – A qualitative user preference study
Source: PLoS One. 2025 Mar 3;20(3):e0318876. doi: 10.1371/journal.pone.0318876 (PMC11875348; doi:10.1371/journal.pone.0318876)
Supplement: S5 Appendix — (DOCX) [file pone.0318876.s005.docx]

**QUESTIONNAIRE**

*(Translated from Norwegian)*

This questionnaire will be used in conjunction with your participation in the research project DiaMestring – pre-study. The information provided in this questionnaire will not be used for other purposes than those informed about in the consent form you have read and signed.

We ask you not to provide name, address, date of birth, or other sensitive information (health information etc.) in this questionnaire.

Terms

*Pre-diabetes* is defined as blood glucose levels beyond normal values, for instance if you have measured high blood sugar at your general practitioner or was told that you have an increased risk of developing type 2 diabetes.

*E-health* is a collective term encompassing ICT use in the health care system. The goal is improvements in quality, security, and efficiency in healthcare by using information technology.

*E-health tools* could be websites or apps used to follow up patients/users, conduct health conversations/consultations, make lifestyle changes and much more.

Please answer as honestly and comprehensively as possible on the questions below. If something is unclear or difficult to understand you may ask us and we will assist you. You may add comments/thoughts you may have at the end (does not necessarily have to be about e-health/e-health tools).

1. What is your participation number?

The same number you were provided with on the name tag during the focus group meeting. Please write a number (not in letters), e.g., 3.

­­­­­_____________

1. What is your age?

- 18-29
- 30-39
- 40-49
- 50-59
- 60-69
- 70-79
- 80 or older

1. What is your gender?

- Male
- Female
- Other

1. Do you have pre-diabetes or type 2 diabetes?

- Pre-diabetes
- Type 2 diabetes

1. What is important for you when you think about your disease and everyday life? Choose all that are important for you.

- Become more active
- Have a more stable blood sugar/regulate my blood sugar in a better way
- Eat better/more varied
- Sleep better
- Weight regulation
- Feel better (better mood, joy, energy etc.)
- Other (write below):

______________________________________________________________________________________________________________________________________________________________________________________________________

1. Which of the following do you think are useful to register and view in an e-health program? Choose all that you think are useful

- Diet
- Sleep
- Physical activity/training
- Body weight
- Blood sugar
- Psychological state (mood etc.)
- Other (write below):

______________________________________________________________________________________________________________________________________________________________________________________________________

1. If there were some that you did **not** choose above, what is the reason you do not consider them as important/useful to register?

E.g., if you did not choose «body weight», is there a reason why you do not consider this as important?

_______________________________________________________________________________________________________________________________________________________________________________________________________________

1. What do you think an e-health program should include for **you** to use it in the long-term? Imagine a future e-health tool for pre-diabetes/type 2 diabetes.

E.g., easy to use, motivating, nice figures, easy to register etc.

_________________________________________________________________________________________________________________________________________________________________________________________________________________________________________________________________________________________________________________________________________________________

1. Do you think communication with a coach is important for you? E.g., follow-up conversations, feedback …

__________________________________________________________________________________________________________________________________________

1. What other information would you like to share with your coach? Choose all that are important to you.

- Diet
- Weight change
- Physical activity
- Medication use
- Psychological state
- Blood sugar data
- Other (write below):

_______________________________________________________________________________________________________________________________________________________________________________________________________________

1. How can sharing information with your coach impact your self-management of your disease?

_________________________________________________________________________________________________________________________________________________________________________________________________________________________________________________________________________________________________________________________________________________________

1. How often would you like to receive digital follow-up with your coach through the e-health program?
   Choose one of the alternatives below.

- Weekly
- Each quarter
- Monthly
- More rarely
- Other (write below):

__________________________________________________________________________________________________________________________________________

1. Do you have other thoughts about e-health or the use of e-health tools in prevention, treatment, and remission of lifestyle-related diseases?

_________________________________________________________________________________________________________________________________________________________________________________________________________________________________________________________________________________________________________________________________________________________

1. Do you have other comments or things you would like to say something about?

_________________________________________________________________________________________________________________________________________________________________________________________________________________________________________________________________________________________________________________________________________________________
